# Organisational Factors Induce Prolonged Emergency Department Length of Stay in Elderly Patients – A Retrospective Cohort Study

**DOI:** 10.1371/journal.pone.0135066

**Published:** 2015-08-12

**Authors:** Steffie H. A. Brouns, Patricia M. Stassen, Suze L. E. Lambooij, Jeanne Dieleman, Irene T. P. Vanderfeesten, Harm R. Haak

**Affiliations:** 1 Department of Internal Medicine, Máxima Medical Centre, Eindhoven/Veldhoven, the Netherlands; 2 Department of Internal Medicine, division of general medicine, section acute medicine, Maastricht University Medical Centre, Maastricht, the Netherlands; 3 Máxima Medical Centre Academy, Máxima Medical Centre, Eindhoven/Veldhoven, the Netherlands; 4 School of Industrial Engineering, Eindhoven University of Technology, Eindhoven, the Netherlands; 5 Maastricht University, Department of Health Services Research and CAPHRI School for Public Health and Primary Care, Maastricht, the Netherlands; University of Florida, UNITED STATES

## Abstract

**Study objective:**

To assess the association of patient and organisational factors with emergency department length of stay (ED-LOS) in elderly ED patients (226565 years old) and in younger patients (<65 years old).

**Methods:**

A retrospective cohort study of internal medicine patients visiting the emergency department between September 1^st^ 2010 and August 31^st^ 2011 was performed. All emergency department visits by internal medicine patients 226565 years old and a random sample of internal medicine patients <65 years old were included. Organisational factors were defined as non-medical factors. ED-LOS is defined as the time between ED arrival and ED discharge or admission. Prolonged ED-LOS is defined as ≥75^th^ percentile of ED-LOS in the study population, which was 208 minutes.

**Results:**

Data on 1782 emergency department visits by elderly patients and 597 emergency department visits by younger patients were analysed. Prolonged ED-LOS in elderly patients was associated with three organisational factors: >1 consultation during the emergency department visit (odds ratio (OR) 3.2, 95% confidence interval (CI) 2.3–4.3), a higher number of diagnostic tests (OR 1.2, 95% CI 1.16–1.33) and evaluation by a medical student or non-trainee resident compared with a medical specialist (OR 4.2, 95% CI 2.0–8.8 and OR 2.3, 95% CI 1.4–3.9). In younger patients, prolonged ED-LOS was associated with >1 consultation (OR 2.6, 95% CI 1.4–4.6). Factors associated with shorter ED-LOS were arrival during nights or weekends as well as a high urgency level in elderly patients and self-referral in younger patients.

**Conclusion:**

Organisational factors, such as a higher number of consultations and tests in the emergency department and a lower seniority of the physician, were the main aspects associated with prolonged ED-LOS in elderly patients. Optimisation of the organisation and coordination of emergency care is important to accommodate the needs of the continuously growing number of elderly patients in a better way.

## Background

The Emergency Department (ED) manages complex patient populations and is under continuous time pressure [1,2]. The increase in the number of ED visits over the past decade has resulted in ED crowding [3–5]. In particular, the substantial growth of ED visits by elderly patients (≥ 65 years old) has placed a heavy burden on the acute care system [6–9].

ED crowding leads to prolonged emergency department length of stay (ED-LOS), delay in treatment and a worse medical outcome, such as a longer hospital stay and a higher mortality rate [3,5,8,10–12]. In addition, prolonged ED-LOS reduces patient satisfaction and has a negative impact on the quality of care and the adherence to ED guidelines [3,13]. Therefore, ED-LOS is marked as an important quality indicator of emergency care [14–18]. The association of prolonged ED-LOS with poor patient outcome has been studied in various settings and patient groups, demonstrating diverse results [19–22]. As to whether the patients’ age has an effect on ED-LOS remains unclear from previously published reports. ED-LOS has been reported to exceed 4 hours in 26% of elderly patients and in 11% of patients aged 18–64 years [23]. In contrast, a large prospective study showed no association between prolonged ED-LOS and age [24].

Elderly patients represent a complex population in the ED, owing to a sometimes atypical presentation and to the presence of multi-morbidity [25]. In addition, they often have high urgency problems and are frequently transported by ambulance [26,27]. In elderly patients, an ED visit may prelude functional decline. The average 30-day mortality rate of elderly patients following an ED visit is 10% [25,28]. Given the anticipated increasing size of this vulnerable population presenting to the ED, it is necessary to avoid prolonged ED-LOS in order to maintain and improve patient outcome and quality of care at the ED. Our hypotheses are that ED-LOS is mainly influenced by organisational factors instead of disease-related factors and that the influence of organisational factors on prolonged ED-LOS is more prominent in elderly patients than in younger patients (<65 years old).

The objectives of this study are to assess the association of medical factors and organisational factors with ED-LOS in elderly patients and to explore the effect of age on predicting factors of prolonged ED-LOS.

## Methods

### Study design, setting and participants

Exemption of approval by the Institutional Review Board of Máxima Medical Centre (MMC) was acquired. A retrospective cohort study was conducted at MMC, a 550-bed teaching hospital in the Netherlands. Approximately 28,000 patients visit the ED of Máxima Medical Centre annually, of which 13–14% requires assessment by an internist. Most patients for internal medicine are referred to the ED by a general practitioner (GP, who provide service 24/7) (51.8%), while others are referred by ambulance (10.9%), are referred by medical specialists (8.5%), or are self-referrals (28.8%). Either a medical student in the last year of medical education, a non-trainee resident (physicians who have not yet started traineeship in a clinical speciality), a trainee resident or an emergency physician will assess the patients presenting to the ED, supervised by an internist [29].

Data on all ED visits of patients ≥ 65 years old, referred to the ED for internal medicine between September 1^st^ 2010 and August 31^st^ 2011, were extracted according to a fixed data collection form by one investigator. Given the retrospective observational design, no informed consent was obtained, as patient data was anonymized and deidentified during data extraction and prior to statistical analysis, and accessing of data by the authors (excluding SB). The authors may have had interactions with the patients used in the study (SB, SL, and HH), which was considered in the waiver of approval provided by the Ethics Committee of MMC. A sample of patients < 65 years old, presenting to the ED for internal medicine, was randomly obtained from the ED visit list by the random SPSS procedure (IBM SPSS Statistics for Windows, Version 19.0, Armonk, New York). The sample population of younger patients was comparable with the total population of younger patients in terms of age, gender, day of ED presentation, time of ED presentation, mode of presentation, triage category and final disposition, as visually checked with descriptive statistics and tested using Chi-square and unpaired T-tests. Exclusion criteria were an overt incorrect ED recording time or a principle treating specialty in the ED other than internal medicine.

### Data sources and variables

Baseline and medical data were retrospectively retrieved from electronic patient and hospital records using standard data-collection forms. Data included age, gender, medical history, medication use, ED visits and hospitalisation in the previous three months, triage level, presenting complaint and ED diagnosis. Organisational factors, defined as non-medical factors, included day of the week and time of ED visit, mode of presentation, seniority of the first physician who assessed/treated the patient in the ED, number and type of diagnostic tests, number of consultations by medical specialties other than internal medicine at the ED, medical procedures performed in the ED and medication administered during the ED stay. In addition, we retrieved ED recording times, final disposition, date of admission and discharge, and date of last follow-up and date of death.

### Definitions

In absence of a definition of prolonged ED-LOS in the literature, prolonged ED-LOS was defined as a length of stay that lasted longer than the 75^th^ percentile of ED-LOS in the total study population (elderly and younger patients), which was an ED-LOS ≥ 208 minutes. Medical history and presenting complaint, as documented in the patient ED records, were classified according to the International Classification of Disease-10 (ICD-10). The ICD-10 category “Symptoms, signs and abnormal clinical and laboratory findings, not elsewhere classified” was classified as aspecific complaints. The following ICD-10 categories were combined into the group “miscellaneous”: diseases of the musculoskeletal system, genitourinary system, eye and adnexa, ear and mastoid process, skin and subcutaneous tissue, injury or poisoning, and external causes of morbidity and mortality (See S1 Table). The Charlson Co-morbidity index (CCI) was calculated to assess the comorbidity levels of the patients [30]. Polypharmacy was defined as the use of five or more different medications [31]. Time of presentation was classified as morning (7.00–11.59 h), afternoon (12.00–16.59 h), evening (17.00–23.59 h) and night (0.00–6.59 h). Mode of presentation was categorised into referral by a GP, ambulance or medical specialist and self-referral. ED recording times (in minutes) were sectioned into 1) time in waiting room: time from ED arrival to ED bed placement, 2) time to triage: time from ED arrival to assignment of a triage category, 3) treatment time: time from ED bed placement to final disposition and 4) ED-LOS: time between ED arrival and ED discharge or hospital admission. Triage at presentation was performed using the Manchester Triage System (MTS) [32]. Urgency levels were classified as high (MTS categories red and orange), moderate (MTS category yellow) and standard (MTS category green (no patients are assigned to MTS category blue at the ED)). The seniority of the first physician was classified as medical student in last year of medical education, non-trainee resident, trainee resident or medical specialist (internist or emergency physician) [33]. Diagnostic tests performed at the ED comprised of a blood test, an arterial blood-gas test, a urine test, a culture test, an electrocardiogram (ECG), an X-ray, ultrasonography, computed tomography (CT) scan or magnetic resonance imaging (MRI). Medical procedures consisted of intubation, placement of urinary catheter or gastric tube, cardiac rhythm monitoring and administration of oxygen. Prolonged hospital LOS was defined as a stay that lasted longer than the 75^th^ percentile of hospital LOS from ED discharge until hospital discharge, as calculated for all patients that were hospitalised, which was ≥ 12 days.

### Statistical analysis

Statistical analysis was performed using SPSS version 19.0. Comparisons of the characteristics of patients ≥ 65 years old and patients < 65 years old were tested using the Chi-square test for categorical variables. The numerical variables were tested using one-way analysis of variance (ANOVA), the Kruskal-Wallis test, Mann-Whitney U-test, and unpaired T-test, depending on the number of groups and the distribution pattern of the variable. Missing data were categorised as “unknown” and included in the analyses to assess the influence of missing data on ED-LOS. Univariable and multivariable logistic regression analyses were performed in order to estimate the effect of various factors on prolonged ED-LOS and to calculate the odds ratio (OR) with 95% confidence intervals (CI). Multivariable analysis was done to calculate adjusted OR (ORadj) and included all variables from the univariable analysis associated with prolonged ED-LOS with a p-value of ≤ 0.05. A two-sided p-value < 0.05 was considered statistical significant.

## Results

### Study population

**Fig 1:**
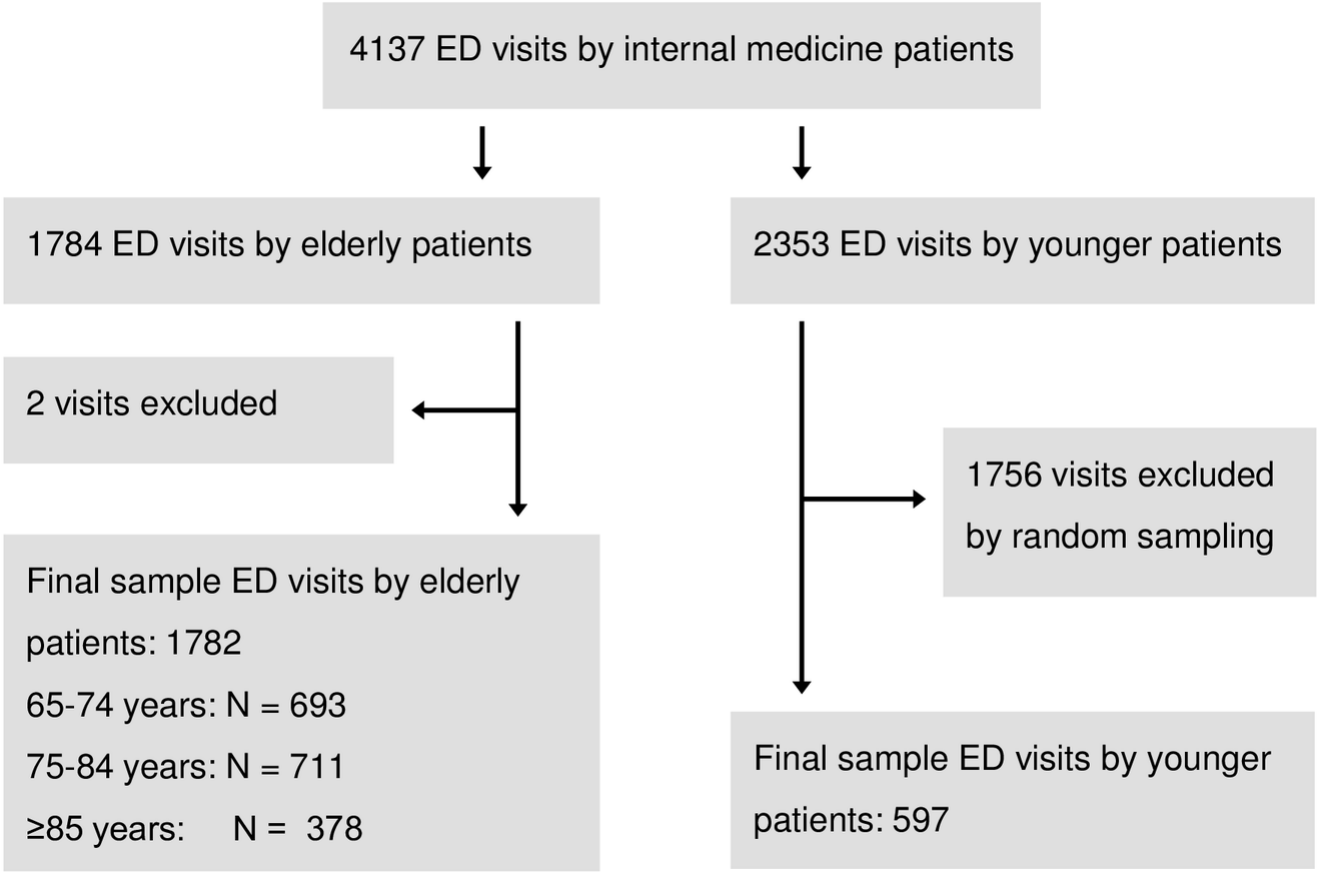
Flow chart of the studied population. ED = emergency department.

In the study period, 4137 ED visits by internal medicine patients were recorded, of which 1784 visits (43.1%) were made by 1435 elderly patients (Fig 1). Two ED visits by elderly patients were excluded because of inaccurate ED recording times. In the same period, 2353 ED visits were by younger patients, of which 597 (25%) visits made by 564 patients were randomly selected (Fig 1).

#### Baseline characteristics and medical factors

The sex distribution was similar for both the elderly and younger patients visiting the ED (Table 1). Elderly patients had more comorbidity (CCI 2.5 vs. 1.0, p < 0.001) and more prevalent polypharmacy (57.7 vs. 17.1%, p < 0.001) than younger patients (Table 1). They also had visited the ED (28.3 vs. 21.1%, p < 0.001) more often and were hospitalised (28.1 vs. 14.2%, p < 0.001) more often in the three months before the ED visit than younger patients. In total, 1100 (61.7%) elderly patients presented with aspecific complaints, of whom 11.0% had a high urgency level, 57.6% moderate, 30.7% standard, and 0.6% were not classified. In younger patients with aspecific complaints (65.3%), 6.4% had a high urgency level, 54.1% moderate, 36.9% standard, and 2.6% were not classified.

**Table 1:** Characteristics of ED visits by internal medicine patients.

Characteristic	ED visit by elderly patients (n = 1782)	ED visit by younger patients (n = 597)
Mean (SD) age, years	77.5 (7.7)	43.5 (14)
No. of male participants (%)	824 (46.2)	281 (47.1)
Mean (SD) CCI**	2.5 (2.2)	1.0 (1.7)
Polypharmacy (%)**	1028 (57.7)	102 (17.1)
No. of ED visit in previous 3 months (%)**	505 (28.3)	126 (21.1)
No. of admissions in previous 3 months (%)**	501 (28.1)	85 (14.2)
No. of patients per day of presentation		
Weekday (%) *	1386 (77.8)	427 (71.5)
Weekend (%) *	396 (22.2)	170 (28.5)
No. of patients per time of presentation		
Morning (%)**	368 (20.6)	117 (19.6)
Afternoon (%)**	750 (42.0)	195 (32.7)
Evening (%)**	532 (29.9)	193 (32.3)
Night (%)**	132 (7.4)	92 (15.4)
No. of patients per mode of presentation		
GP referral (%)**	1270 (71.3)	228 (38.2)
Medical specialist (%)**	153 (8.6)	59 (9.9)
Ambulance (%)**	158 (8.9)	70 (11.7)
Self-referral (%)**	201 (11.3)	240 (40.2)
Urgency level		
High (%)**	207 (11.6)	59 (9.9)
Moderate (%)**	960 (53.9)	311 (52.1)
Standard (%)**	603 (33.8)	193 (32.3)
No triage (%)**	12 (0.7)	34 (5.7)
Seniority of first physician on ED		
Medical student (%)**	51 (2.9)	9 (1.5)
Non-trainee resident (%)**	558 (31.3)	175 (29.3)
Trainee resident (%)**	1039 (58.3)	326 (54.6)
Medical specialist (%)**	110 (6.2)	63 (10.6)
Unknown (%)**	24 (1.3)	24 (4.0)
Mean no. (SD) diagnostic tests on ED**	3.2 (1.8)	2.1 (1.6)
Medication on ED (%)*	762 (42.8)	214 (35.9)
Medical procedures on ED (%)**	746 (41.9)	158 (26.5)
No. of admissions (%)**	1299 (72.9)	231 (38.7)

P-values were calculated using unpaired T-test and Chi-square test. SD = standard deviation; ED = emergency department; mo = months; CCI = Charlson co-morbidity index.

* = 0.001 < p < 0.05

** = p < 0.001.

#### Organisational factors

Elderly patients were referred by their GP in 71.3% of cases compared with only 38.2% in younger patients (Table 1). In both age groups, most patients presented to the ED on Friday (16.4% in elderly and 17.6% in younger patients). The diagnostic work-up in the ED was more extensive for elderly patients (mean: 3.2 tests in elderly patients vs. 2.1 in younger patients, p < 0.001). Elderly patients more often received medication (42.8 vs. 35.9%, respectively, p = 0.011) and underwent medical procedures (41.9 vs. 26.5%, respectively, p < 0.001) at the ED than younger patients. In 72.9% of elderly patients, the ED visit resulted in hospital admission, compared with 38.7% of younger patients (p < 0.001). If hospitalised, the median hospital LOS was 6 days in elderly patients (range 1–91) and 3 days (range 1–89) in younger patients (p < 0.001).

### ED recording times

**Fig 2:**
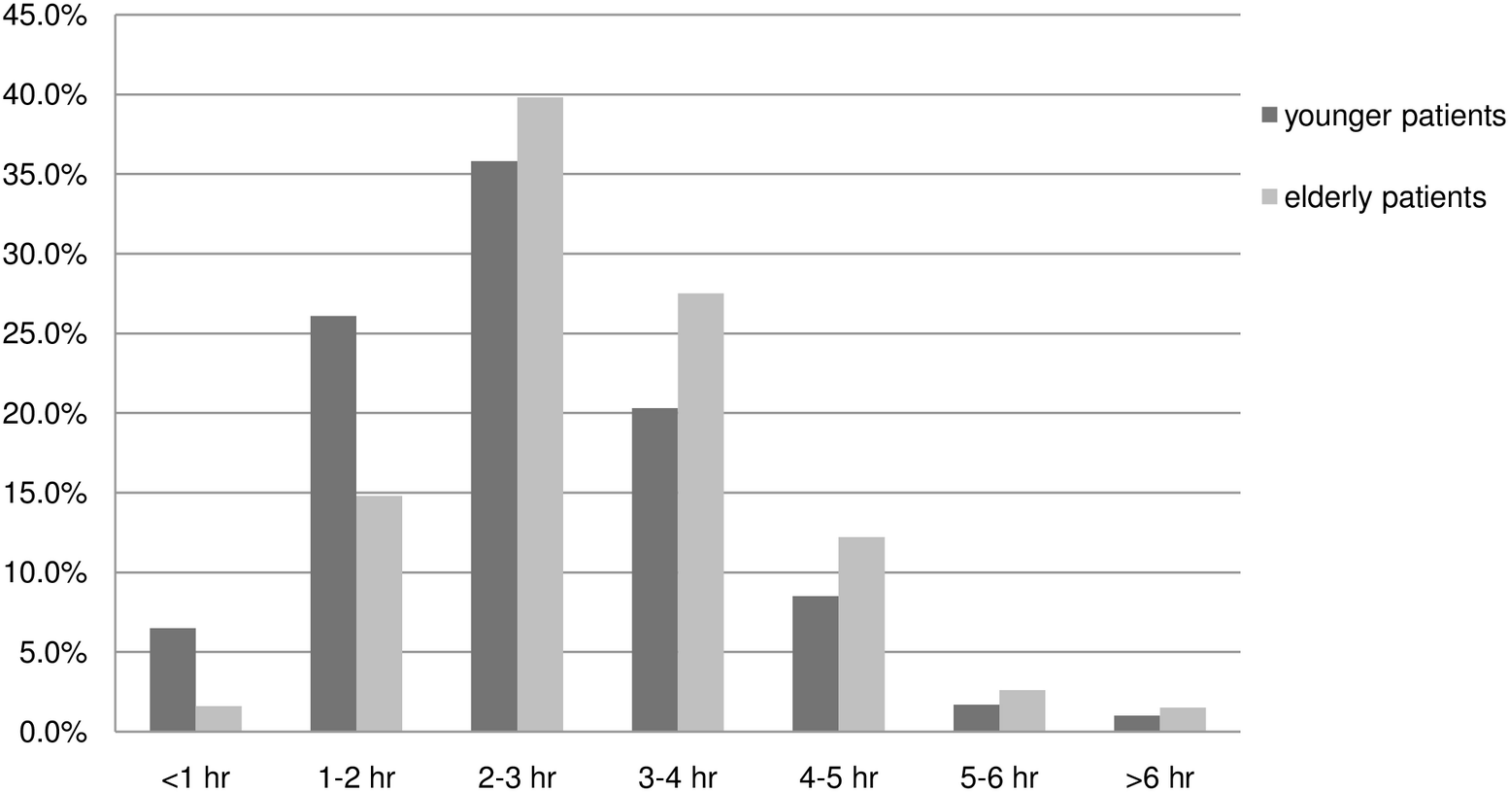
Emergency department length of stay (ED-LOS) per hour for elderly patients and younger patients.

The median ED-LOS was 172 minutes in elderly patients (range 6–542), compared with 147 minutes for younger patients (range 3–413, p < 0.001) (Fig 2). Among elderly patients, 27.1% experienced a prolonged ED-LOS (i.e. ED-LOS ≥ 208 min), compared with 20.3% for younger patients (p = 0.002). The ED-LOS exceeded 4 hours in 16.3% of elderly patients and 11.2% of patients <65 years (p = 0.003). The median treatment time for elderly patients was 158 minutes versus 130 minutes for younger patients (p < 0.001). Both the time spent in the waiting room (5 vs. 2 minutes, p<0.001) and time to triage (10 vs. 9 minutes, p = 0.011) were longer in elderly patients than in younger patients.

### Determinants of prolonged ED-LOS

#### Baseline characteristics and medical factors

In the elderly, a moderate urgency level (compared with a standard urgency), was associated with prolonged ED-LOS (OR 1.3, 95% CI 1.01–1.59). Unknown medical history (versus no medical history) was associated with a lower risk of prolonged ED-LOS (OR 0.2, 95% CI 0.1–0.9) (Table 2). Over a quarter (27.6%) of elderly patients with aspecific complaints had a prolonged ED-LOS. The triage level distribution in those patients was similar to those without prolonged ED-LOS. The presence of cognitive impairment (e.g. dementia or delirium) was not associated with prolonged ED-LOS in elderly patients (OR 1.3, 95% CI 0.9–1.8), nor was the presence of polypharmacy or the presenting complaint (Table 2).

**Table 2:** Unadjusted data on the impact of baseline characteristics and medical factors on ED-LOS.

	Elderly patients			Younger Patients		
	ED-LOS <208 min (n = 1299)	ED-LOS ≥ 208 min (n = 483)	OR (95% CI)	ED-LOS < 208 min (n = 474)	ED-LOS 2265 208 min (n = 123)	OR (95% CI)
Female vs. male (%)	692 (53.3)	266 (55.1)	1.1 (0.9–1.3)	250 (52.7)	66 (53.7%)	1.0 (0.7–1.5)
Medical history						
Cognitive impairment (%)	128 (9.9)	59 (12.2)	1.3 (0.9–1.8)	4 (0.8)	-	-
Unknown medical history (%)	25 (1.9)	2 (0.4)	0.2 (0.1–0.9)*	58 (12.2)	10 (8.1)	0.6 (0.3–1.3)
Mean (SD) CCI	2.46 (2.2)	2.45 (2.2)	1.0 (0.95–1.05)	0.97 (1.7)	0.96 (1.7)	1.0 (0.9–1.1)
Polypharmacy						
Yes vs. no (%)	727 (56.0)	301 (62.3)	1.3 (0.99–1.59)	71 (15.0)	31 (25.2)	1.7 (1.1–2.9)*
Unknown vs. no (%)	167 (12.9)	48 (9.9)	0.9 (0.6–1.3)	99 (20.9)	16 (13.0)	0.6 (0.4–1.2)
Previous ED visit						
Yes vs. no visit (%)	378 (29.1)	127 (26.3)	0.9 (0.7–1.1)	99 (20.9)	27 (22.0)	1.0 (0.6–1.7)
Unknown vs. no visit (%)	15 (1.2)	2 (0.4)	0.3 (0.8–1.5)	14 (3.0)	2 (1.6)	0.5 (0.1–2.5)
Previous admission						
Yes vs. no admission (%)	378 (29.1)	123 (25.5)	0.82 (0.65–1.04)	60 (12.7)	25 (20.3)	1.71 (1.02–2.87)*
Unknown vs. no admission (%)	13 (1.0)	-	-	15 (3.2)	1 (0.8)	-
Presenting complaint						
Aspecific complaints (%)	797 (61.4)	303 (62.7)	Reference	299 (63.1)	91 (74.0)	Reference
Endocrine/metabolic (%)	84 (6.5)	25 (5.2)	0.9 (0.5–1.3)	13 (2.7)	2 (1.6)	0.5 (0.1–2.3)
Circulatory (%)	82 (6.3)	31 (6.4)	1.0 (0.6–1.5)	22 (4.6)	7 (5.7)	1.0 (0.4–2.5)
Gastrointestinal (%)	54 (4.2)	22 (4.6)	1.1 (0.6–1.8)	12 (2.5)	4 (3.3)	1.1 (0.3–3.5)
Neoplasm/hematologic (%)	43 (3.3)	16 (3.3)	1.0 (0.5–1.8)	4 (0.8)	1 (0.8)	-
Respiratory (%)	39 (3.0)	13 (2.7)	0.9 (0.5–1.7)	6 (1.3)	1 (0.8)	-
Infectious (%)	38 (2.9)	8 (1.7)	0.6 (0.3–1.2)	7 (1.5)	1 (0.8)	-
Psychiatric/neurologic (%)	34 (2.6)	14 (2.9)	1.1 (0.6–2.0)	3 (0.6)	1 (0.8)	-
“Miscellaneous” complaints (%)	128 (9.9)	51 (10.6)	1.0 (0.7–1.5)	108 (22.8)	15 (12.2)	0.5 (0.3–0.8)*
Urgency level						
High (%)	167 (12.9)	40 (8.3)	0.7 (0.48–1.04)	51 (10.8)	8 (6.5)	0.6 (0.3–1.4)
Moderate (%)	671 (51.7)	289 (59.8)	1.3 (1.01–1.59)*	239 (50.4)	72 (58.5)	1.2 (0.8–1.9)
Standard (%)	450 (34.6)	153 (31.7)	Reference	154 (32.5)	39 (31.7)	Reference
No triage (%)	11 (0.8)	1 (0.2)	-	30 (6.3)	4 (3.3)	0.5 (0.2–1.6)

ED-LOS = Emergency Department Length Of Stay; min = minutes; OR = Odds Ratio; CI = Confidence Interval; CCI = Charlson Comorbidity Index; SD = standard deviation.

* = 0.001 < p < 0.05.

In younger patients, polypharmacy (OR 1.7, 95% CI 1.1–2.9), previous hospital admission (OR 1.7, 95% CI 1.02–2.87), and a presenting complaint classified as “miscellaneous” (OR 0.5, 95% CI 0.3–0.8) rather than aspecific complaints were associated with prolonged ED-LOS (Table 2). In younger patients with aspecific complaints, 23.4% had a prolonged ED-LOS, of which 63.7% had a moderate urgency level and 31.9% a standard.

#### Organisational factors

Risk of prolonged ED-LOS was lower for elderly patients who arrived over the weekend compared with those arriving on a weekday (OR 0.7, 95% CI 0.5–0.9). Similarly, the risk of prolonged ED-LOS was lower during the night (OR 0.4, 95% CI 0.2–0.6) than the morning. The risk was also lower if the patient was referred by a medical specialist (OR 0.66, 95% CI (0.44–0.99) or self-referral (OR 0.6, 95% CI 0.4–0.9), compared with referral by a GP (Table 3).

**Table 3:** Unadjusted data on the impact of organisational factors on ED-LOS.

	Elderly patients			Younger patients		
	ED-LOS < 208 min (n = 1299)	ED-LOS ≥ 208 min (n = 483)	OR (95% CI)	ED-LOS < 208 min (n = 474)	ED-LOS ≥ 208 min (n = 123)	OR (95% CI)
Weekend vs. week (%)	312 (24.0)	84 (17.4)	0.7 (0.5–0.9)*	134 (28.3)	36 (29.3)	1.1 (0.7–1.6)
Time of presentation						
Morning (%)	257 (19.8)	106 (21.9)	Reference	94 (19.8)	26 (21.1)	Reference
Afternoon (%)	513 (39.5)	228 (47.2)	1.1 (0.8–1.4)	148 (31.2)	52 (42.3)	1.3 (0.7–2.2)
Evening (%)	421 (32.4)	133 (27.5)	0.8 (0.57–1.03)	163 (34.4)	35 (28.5)	0.8 (0.4–1.4)
Night (%)	108 (8.3)	16 (3.3)	0.4 (0.2–0.6)**	69 (14.6)	10 (8.1)	0.5 (0.2–1.2)
Mode of presentation						
General practitioner (%)	898 (69.1)	372 (77.0)	Reference	162 (34.2)	66 (53.7)	Reference
Medical specialist (%)	120 (9.2)	33 (6.8)	0.66 (0.44–0.99)*	48 (10.1)	11 (8.9)	0.6 (0.3–1.2)
Ambulance (%)	122 (9.4)	36 (7.5)	0.7 (0.5–1.1)	60 (12.7)	10 (8.1)	0.4 (0.2–0.9)*
Self-referral (%)	159 (12.2)	42 (8.7)	0.6 (0.4–0.9)*	204 (43.0)	36 (29.3)	0.4 (0.3–0.7)**
>1 vs. 1 consultation on ED (%)	127 (9.8)	109 (22.6)	2.7 (2.0–3.6)**	42 (8.9)	25 (20.3)	2.6 (1.5–4.5)**
Seniority of physician on ED						
Medical student (%)	26 (2.0)	25 (5.2)	3.6 (1.8–7.4)**	7 (1.5)	2 (1.6)	2.3 (0.4–13.3)
Non-trainee resident (%)	382 (29.4)	176 (36.4)	1.7 (1.1–2.9)*	134 (28.3)	41 (33.3)	2.4 (1.04–5.79)*
Trainee resident (%)	780 (60.0)	259 (53.6)	1.3 (0.8–2.0)	255 (53.8)	72 (58.5)	2.3 (0.99–5.17)
Medical specialist (%)	87 (6.7)	23 (4.8)	Reference	56 (11.8)	7 (5.7)	Reference
Unknown (%)	24 (1.8)	-	-	22 (4.6)	1 (0.8)	-
No of diagnostic tests on ED (SD)	3.01 (1.8)	3.58 (1.6)	1.2 (1.1–1.3)**	2.01 (1.5)	2.67 (1.6)	1.3 (1.1–1.5)**
Medical procedures at ED (%)	545 (42.0)	201 (41.6)	1.0 (0.8–1.2)	125 (26.4)	33 (26.8)	1.0 (0.7–1.6)
Medication at ED (%)	539 (41.5)	223 (46.2)	1.2 (0.98–1.50)	166 (35.1)	48 (39.0)	1.2 (0.8–1.8)
Admissions (%)	922 (71.0)	377 (78.1)	1.5 (1.1–1.9)*	169 (35.7)	62 (50.4)	1.8 (1.2–2.7)*

ED-LOS = emergency department length of stay; min = minutes; OR = odds ratio; CI = Confidence interval.

* = 0.001 < p < 0.05

** = p < 0.001.

In both elderly and younger patients, more diagnostic tests performed on the ED (OR 1.2, 95% CI 1.1–1.3 and OR 1.3, 95% CI 1.1–1.5), > 1 consultation (OR 2.7, 95% CI 2.0–3.6 and OR 2.6, 95% CI 1.5–4.5) and lower seniority of physician (OR 1.7, 95% CI 1.1–2.9 and OR 2.4, 95% CI 1.04–5.79 when evaluated by a non-trainee resident) at the ED were associated with prolonged ED-LOS. Younger patients arriving by ambulance or after self-referral had a shorter ED-LOS (OR 0.4, 95% CI 0.2–0.9 and OR 0.4, 95% CI 0.3–0.7, respectively).

### Multivariable analysis of factors associated with prolonged ED-LOS

The only baseline or medical characteristic associated with prolonged ED-LOS in elderly patients after multivariable adjustment for other variables was a high urgency level (ORadj 0.4, 95% CI 0.2–0.6) versus a standard urgency level (Table 4).

**Table 4:** Medical and organisational factors with significant impact on ED-LOS after multivariable analysis.

	Elderly patients	Younger patients
	ORadj (95% CI)	ORadj (95% CI)
High urgency level	0.4 (0.2–0.6)**	-
Weekend arrival	0.7 (0.5–0.9)*	-
Arrival during night time	0.4 (0.2–0.7)*	-
Self-referral	-	0.59 (0.35–0.99)*
>1 consultation on ED	3.1 (2.3–4.2)**	2.6 (1.5–4.7)*
Seniority of first physician on ED		
Medical student	4.2 (2.0–8.8)**	-
Non-trainee resident	2.3 (1.4–3.9)*	-
Mean no. of diagnostic tests on ED	1.2 (1.1–1.3)*	-

ED-LOS = emergency department length of stay; ORadj = adjusted odds ratio; CI = confidence Interval.

* = 0.001 < p < 0.05

** = p < 0.001.

However, several organisational factors were associated with prolonged ED-LOS in elderly patients, including, > 1 consultation (ORadj 3.1, 95% CI 2.3–4.2), the number of diagnostic tests (ORadj 1.2, 95% CI 1.1–1.3) and evaluation by a medical student or non-trainee resident (ORadj 4.2, 95% CI 2.0–8.8 and ORadj 2.3, 95% CI 1.4–3.9, respectively) (Table 4). Weekend or night-time arrivals were associated with shorter ED-LOS in elderly patients (ORadj 0.7, 95% CI 0.5–0.9 and ORadj 0.4, 95% CI 0.2–0.7, respectively).

In younger patients, the number of consultations and self-referral remained associated with prolonged ED-LOS in the multivariable analysis (ORadj 2.6, 95% CI 1.5–4.7 and ORadj 0.6, 95% CI 0.35–0.99, respectively) (Table 4).

### Correlation between hospital admission, placement and ED-LOS

The median ED-LOS for elderly patients discharged home was shorter than for those who were hospitalised (158 and 175 minutes, respectively, p < 0.001). The ED-LOS was comparable for both elderly and younger patients if admitted to an ICU (intensive care unit) or medium-care unit, as compared with admission to the acute medical unit or general ward (OR 1.2, 95% CI 0.6–2.3 and OR 2.8, 95% CI 0.8–9.8, respectively).

If admitted, the median hospital LOS in elderly patients was longer if ED-LOS was prolonged rather than normal (8 days (range 1–91) vs. 6 days (range 1–74), p < 0.001). In younger patients, as well, the median hospital LOS was longer in patients with prolonged ED-LOS than in those with a normal ED-LOS of 5 days (range 1–41) versus 2 days (range 1–89, p = 0.001). Accordingly, a prolonged hospital LOS (i.e. a hospital LOS ≥ 12 days) occurred more frequently in elderly patients with prolonged ED-LOS than in those with normal ED-LOS (35.0 vs. 24.7%, OR 1.6, 95% CI 1.3–2.1). Similarly, in younger patients with prolonged ED-LOS, a prolonged hospital LOS occurred more frequently than in those with normal ED-LOS (22.6 vs. 10.7%, OR 2.4, 95% CI 1.1–5.3).

## Discussion

In this study, we have demonstrated that the ED-LOS is considerably longer (30 minutes) in elderly internal medicine patients than in younger patients. The effect of organisational factors on the ED-LOS, such as the number of consultations, number of diagnostic tests performed at the ED and the seniority of the physician, is more evident in elderly patients than in the younger group.

The proportion of patients with a prolonged ED-LOS, for research purpose defined as the 75^th^ percentile of ED-LOS (i.e. ED-LOS ≥ 208 minutes), is higher in the elderly. Moreover, 16.3% of the elderly patients have an ED-LOS exceeding 4 hours, which is predominately caused by organisational factors and its effect on the treatment time of elderly patients.

Our study shows a clear relationship between the ED-LOS and organisational factors. In accordance with other studies, the number of investigations performed at the ED significantly increased the ED-LOS for elderly patients [25–27,34]. Although an extensive diagnostic work-up during ED visits is becoming customary, particularly in elderly patients, it has important implications for emergency care processes [35]. Additionally, the number of consultations involved at the ED is an important contributor to prolonged ED-LOS in the elderly, which is consistent with a study by Vegting et al [23]. In our study, almost 50% of elderly patients with multiple consultations at the ED has an ED-LOS exceeding 4 hours, which may reflect the complexity of the elderly population.

Apart from a beneficial effect on ED-LOS of more staffing at the ED, as reported before, [36] the type of physician appears to be of special importance. Our study shows that the lower seniority of the first treating physician at the ED is a significant determinant for the prolonged ED-LOS in elderly patients. This indicates that experience and education of the ED doctors plays an important role in the occurrence prolonged ED-LOS, especially in the elderly [26,27].

Although resources and staffing levels are usually reduced on weekends and during the night, [37–41] our data show a positive association between ED-LOS and temporal factors, such as day or time of ED arrival, in elderly patients. This might be explained by the number of patients presenting at our ED, as this is considerably lower on weekends or during the night, suggesting that the number of patients at the ED may contribute more to the prolonged ED-LOS of elderly patients than the availability of resources or ED personnel [37,39].

A remarkable result is the lack of association between ED-LOS in elderly patients and medical or baseline factors, such as CCI, medication use, and presenting complaint. In addition, the presence of cognitive impairment in elderly patients does not affect ED-LOS. These findings contradict other reviews, in which factors such as comorbidity, atypical presentation and polypharmacy were mentioned to be of major influence in ED evaluation of elderly patients [26,27]. The only medical factor in elderly patients with influence on ED-LOS in our population is a high urgency level, which is associated with a shorter ED-LOS. This is also in contrast with other studies that found prolonged ED-LOS in critically ill patients [14,34,42]. This discrepancy can be explained by the presence of an access block to the ICUs in these studies, caused by the inability to transfer admitted ED patients to ICU beds, which hardly occurs in our hospital.

Another main finding in our study is the difference in impact of organisational factors on ED-LOS between elderly and younger patients. Although several organisational factors contribute to prolonged ED-LOS in the elderly, only multiple consultations in the ED were associated with prolonged ED-LOS in the younger patient group. There is no association between the seniority of the physician, number of tests performed or temporal factors and ED-LOS in younger patients. On the other hand, in younger self-referred patients, the ED-LOS is significantly shorter. These patients typically have no need for diagnostic work-up (25.4%), require only one consultation at the ED (91.2%), and are discharged following the ED visit (80%). Hence, it is predominantly the elderly population that affects the emergency care processes at our ED, as is consistent with other studies [12,24,34,43].

### Limitations

Our findings may have been influenced by several limitations. Firstly, owing to the single-centre setting, our findings may be less applicable to other hospitals and other countries. Although the healthcare system is well organised in the Netherlands, the organisation of emergency care in other countries should be taken into account in interpreting our findings. As a consequence our study may not address some of the problems encountered in other settings. Nevertheless, the findings of our study may very well apply to other settings and explain part of the problems. Secondly, there is a risk of bias, because of the retrospective observational design. It is possible that part of the data were incomplete or incorrect, such as for example ED recording times. However, we have no reason to believe that the resulting misclassification is differential except for extreme short visits, which may have more missing data than longer visits. Random misclassification of determinants may have diluted contrasts. The reference category of the presenting complaints, being the largest group, comprised a range of signs and symptoms, which may have introduced noise. The effect of missing values for the ED-LOS was evaluated by including these in the analyses. Overall, more data was missing in younger patients than in elderly patients, yet missing data was not associated with a shorter ED-LOS. Thirdly, the relatively small number of younger patients may contribute to a reduced reliability of our results, due to lack of power. Fourthly, in the absence of a generally accepted definition of prolonged ED-LOS, we based the definition of prolonged ED-LOS for our study on the upper quartile of recorded times in the entire population studied. Although, the relevance of ED-LOS >208 minutes is uncertain, it is useful for the identification of risk factors. Lastly, the effect of staffing levels of medical personnel as well as radiology and laboratory staff, and their workload on ED-LOS were not included in our analysis.

### Implications

This study emphasises the need for a distinct emergency care approach for elderly patients presenting in the ED. In addition, it suggests that sufficient training of ED doctors for the emergency care of the elderly population can help to assess and treat these patients in a timely and effectively manner [44].

The negative impact of the number of consultations on the ED-LOS provides an important opportunity to improve care, since the waiting time between assessments can be reduced if collaboration between different disciplines can be enhanced. Therefore, coordination of emergency evaluation by a leading physician at the ED, specifically in elderly patients, could be helpful in improving the quality of acute care and in reducing the ED-LOS [23]. In addition, a possible solution to reduce prolonged ED-LOS caused by an extensive diagnostic work-up is the implementation of diagnostic-triage standing orders, which are medical orders developed for distinct types of complaints performed by ED nurses (advanced triage). This has previously shown to be beneficial in reducing ED treatment times [45]. Moreover, the development of a clinical decision rule or care pathway for elderly ED patients could potentially improve efficiency in emergency care and diagnostic processes and, subsequently, reduce ED-LOS. However, the cost effectiveness of such an intervention needs to be considered.

Reorganisation of emergency care processes, following the implementation of the four hour target in the UK, contributed towards an improvement in patient flow and reduced ED-LOS, although the relevance of a specific cut-off of ED-LOS remains questionable [46,47]. However, as the number of elderly patients presenting to the ED is expected to increase, the high percentage of prolonged ED-LOS in this population will have a profound impact on EDs. The introduction of a similar target in the Netherlands may facilitate the required modification of the emergency care system in order to improve the quality of acute care [18,47,48].

The risks associated with a prolonged ED-LOS and a complete evaluation need to be weighed against the benefits of a shorter ED-LOS with possible incomplete evaluation, balancing efficiency with accuracy and optimal care in this vulnerable group. Furthermore, patients that would benefit from a higher degree of expertise and are more susceptible to risks associated with prolonged ED-LOS need to be identified. Future prospective studies could examine the impact of prolonged ED-LOS on the quality of care for the elderly and patient outcome, specifically relevant to this population, such as the occurence of complications, hospital LOS and functional decline.

## Conclusions

ED-LOS was considerably longer in elderly patients than in younger patients in our ED. Prolonged ED-LOS in elderly patients was associated with medical and organisational factors, such as a higher number of tests or consultations involved during the ED visit and the low seniority of the physician. Baseline factors, such as medical history, appeared to be of limited influence on prolonged ED-LOS. These findings indicate that improving operational efficiency and coordination in emergency care processes by focusing on organisational factors, without compromising quality of care, is necessary to better suit the needs of the continuously growing population of elderly patients in the ED.

## Supporting Information

S1 Table Miscellaneous complaints in elderly and younger patients and prolonged ED-LOS. (DOCX)

## References

[1] Bernstein SL, Verghese V, Leung W, Lunney AT, Perez I. Development and validation of a new index to measure emergency department crowding. Acad Emerg Med. 2003;10: 938–942. 12957975 10.1111/j.1553-2712.2003.tb00647.x

[2] Wilber ST, Gerson LW, Terrell KM, Carpenter CR, Shah MN, Heard K, et al Geriatric emergency medicine and the 2006 Institute of Medicine reports from the Committee on the Future of Emergency Care in the U.S. health system. Acad Emerg Med. 2006;13: 1345–1351. 17071799 10.1197/j.aem.2006.09.050

[3] Bernstein SL, Aronsky D, Duseja R, Epstein S, Handel D, Hwang U, et al (2009) The effect of emergency department crowding on clinically oriented outcomes. Acad Emerg Med 16(1): 1–10. 10.1111/j.1553-2712.2008.00295.x 19007346

[4] Bernstein SL, Asplin BR (2006) Emergency department crowding: old problem, new solutions. Emerg Med Clin North Am 24(2): 821–837. 16982341 10.1016/j.emc.2006.06.013

[5] Asplin BR, Magid DJ, Rhodes KV, Solberg LI, Lurie N, Camargo CA Jr. (2003) A conceptual model of emergency department crowding. Ann Emerg Med 42(2): 173–180. 12883504 10.1067/mem.2003.302

[6] Salvi F, Morichi V, Grilli A, Giorgi R, Spazzafumo L, Polonara S, et al (2008) A geriatric emergency service for acutely ill elderly patients: pattern of use and comparison with a conventional emergency department in Italy. J Am Geriatr Soc 56(11): 2131–2138. 10.1111/j.1532-5415.2008.01991.x 19016945

[7] Di Bari M, Balzi D, Roberts AT, Barchielli A, Fumagalli S, Ungar A, et al (2010)Prognostic stratification of older persons based on simple administrative data: development and validation of the "Silver Code," to be used in emergency department triage. J Gerontol A Biol Sci Med Sci 65(2): 159–164. 10.1093/gerona/glp043 19349591

[8] LaCalle E, Rabin E (2010) Frequent users of emergency departments: the myths, the data, and the policy implications. Ann Emerg Med 56(1): 42–48. 10.1016/j.annemergmed.2010.01.032 20346540

[9] Roberts DC, McKay MP, Shaffer A (2008) Increasing rates of emergency department visits for elderly patients in the United States, 1993 to 2003. Ann Emerg Med 51(6): 769–774. 18069088 10.1016/j.annemergmed.2007.09.011

[10] Hwang U, McCarthy ML, Aronsky D, Asplin B, Crane PW, Craven CK, et al (2011) Measures of crowding in the emergency department: a systematic review. Acad Emerg Med 18(5): 527–538. 10.1111/j.1553-2712.2011.01054.x 21569171

[11] Richardson DB (2006) Increase in patient mortality at 10 days associated with emergency department overcrowding. Med J Aust 184(5): 213–216. 16515430 10.5694/j.1326-5377.2006.tb00204.x

[12] Liew D, Liew D, Kennedy MP (2003) Emergency department length of stay independently predicts excess inpatient length of stay. Med J Aust 179(10): 524–526. 14609414 10.5694/j.1326-5377.2003.tb05676.x

[13] Hollander JE, Pines JM (2007) The emergency department crowding paradox: the longer you stay, the less care you get. Ann Emerg Med 50(5): 497–499. 17583380 10.1016/j.annemergmed.2007.05.002

[14] Ding R, McCarthy ML, Desmond JS, Lee JS, Aronsky D, Zeger SL (2010) Characterizing waiting room time, treatment time, and boarding time in the emergency department using quantile regression. Acad Emerg Med 17(8): 813–823. 10.1111/j.1553-2712.2010.00812.x 20670318

[15] Kocher KE, Sklar DP, Mehrotra A, Tayal VS, Gausche-Hill M, Myles Riner R (2010) Categorization, designation, and regionalization of emergency care: definitions, a conceptual framework, and future challenges. Acad Emerg Med 17(12): 1306–1311. 10.1111/j.1553-2712.2010.00932.x 21122012

[16] Rathlev NK, Chessare J, Olshaker J, Obendorfer D, Mehta SD, Rothenhaus T, et al (2007) Time series analysis of variables associated with daily mean emergency department length of stay. Ann Emerg Med 49(3): 265–271. 17224203 10.1016/j.annemergmed.2006.11.007

[17] Singer AJ, Thode HC Jr, Viccellio P, Pines JM (2011) The association between length of emergency department boarding and mortality. Acad Emerg Med 18(12): 1324–1329. 10.1111/j.1553-2712.2011.01236.x 22168198

[18] Weber EJ, Mason S, Carter A, Hew RL (2011) Emptying the corridors of shame: organizational lessons from England's 4-hour emergency throughput target. Ann Emerg Med 57(2): 79–88.e1. 10.1016/j.annemergmed.2010.08.013 21251521

[19] Hwang U, Richardson LD, Sonuyi TO, Morrison RS (2006) The effect of emergency department crowding on the management of pain in older adults with hip fracture. J Am Geriatr Soc 54(2): 270–275. 16460378 10.1111/j.1532-5415.2005.00587.x

[20] Elmer J, Pallin DJ, Liu S, Pearson C, Chang Y, Camargo CA Jr, et al (2012) Prolonged emergency department length of stay is not associated with worse outcomes in patients with intracerebral hemorrhage. Neurocrit Care 17(3): 334–342. 10.1007/s12028-011-9629-1 21912953 PMC3684176

[21] Schull MJ, Vermeulen M, Slaughter G, Morrison L, Daly P. (2004) Emergency department crowding and thrombolysis delays in acute myocardial infarction. Ann Emerg Med 44(6): 577–585. 15573032 10.1016/j.annemergmed.2004.05.004

[22] Chalfin DB, Trzeciak S, Likourezos A, Baumann BM, Dellinger RP, DELAY-ED study group. (2007) Impact of delayed transfer of critically ill patients from the emergency department to the intensive care unit. Crit Care Med 35(6): 1477–1483. 17440421 10.1097/01.CCM.0000266585.74905.5A

[23] Vegting IL, Nanayakkara PW, van Dongen AE, Vandewalle E, van Galen J, Kramer MH, et al (2011) Analysing completion times in an academic emergency department: coordination of care is the weakest link. Neth J Med 69(9): 392–398. 21978983

[24] Casalino E, Wargon M, Peroziello A, Choquet C, Leroy C, Beaune S, et al (2014) Predictive factors for longer length of stay in an emergency department: a prospective multicentre study evaluating the impact of age, patient's clinical acuity and complexity, and care pathways. Emerg Med J. 31(5): 361–368. 10.1136/emermed-2012-202155 23449890

[25] Aminzadeh F, Dalziel WB (2002) Older adults in the emergency department: a systematic review of patterns of use, adverse outcomes, and effectiveness of interventions. Ann Emerg Med 39(3): 238–247. 11867975 10.1067/mem.2002.121523

[26] Salvi F, Morichi V, Grilli A, Giorgi R, De Tommaso G, Dessì-Fulgheri P (2007) The elderly in the emergency department: a critical review of problems and solutions. Intern Emerg Med 2(4): 292–301. 18043874 10.1007/s11739-007-0081-3

[27] Samaras N, Chevalley T, Samaras D, Gold G (2010) Older patients in the emergency department: a review. Ann Emerg Med 56(3): 261–269. 10.1016/j.annemergmed.2010.04.015 20619500

[28] Hastings SN, Purser JL, Johnson KS, Sloane RJ, Whitson HE (2008) Frailty predicts some but not all adverse outcomes in older adults discharged from the emergency department. J Am Geriatr Soc 56(9): 1651–1657. 10.1111/j.1532-5415.2008.01840.x 18691282 PMC2676906

[29] Holmes JL (2010) Emergency medicine in the Netherlands. Emerg Med Australas 22(1): 75–81. 10.1111/j.1742-6723.2009.01259.x 20152006

[30] Needham DM, Scales DC, Laupacis A, Pronovost PJ (2005) A systematic review of the Charlson comorbidity index using Canadian administrative databases: a perspective on risk adjustment in critical care research. J Crit Care 20(1): 12–19. 16015512 10.1016/j.jcrc.2004.09.007

[31] Gnjidic D, Hilmer SN, Blyth FM, Naganathan V, Waite L, Seibel MJ, et al (2012) Polypharmacy cutoff and outcomes: five or more medicines were used to identify community-dwelling older men at risk of different adverse outcomes. J Clin Epidemiol 65(9): 989–995. 10.1016/j.jclinepi.2012.02.018 22742913

[32] Mackway-Jones K. Manchester Triage Group. (2005) Emergency Triage. 2nd ed: Bmj Publishing Group 192p.

[33] Thijssen WA, Giesen PH, Wensing M. (2012) Emergency departments in The Netherlands. Emerg Med J 29(1): 6–9. 10.1136/emermed-2011-200090 22036937

[34] Herring A, Wilper A, Himmelstein DU, Woolhandler S, Espinola JA, Brown DF, et al (2009) Increasing length of stay among adult visits to U.S. Emergency departments, 2001–2005. Acad Emerg Med 16(7): 609–616. 10.1111/j.1553-2712.2009.00428.x 19538503

[35] Kocher KE, Meurer WJ, Desmond JS, Nallamothu BK. (2012) Effect of testing and treatment on emergency department length of stay using a national database. Acad Emerg Med 19(5): 525–534. 10.1111/j.1553-2712.2012.01353.x 22594356

[36] Bucheli B, Martina B. (2004) Reduced length of stay in medical emergency department patients: a prospective controlled study on emergency physician staffing. Eur J Emerg Med 11(1): 29–34. 15167190 10.1097/00063110-200402000-00006

[37] Barba R, Losa JE, Velasco M, Guijarro C, Garcia de Casasola G, Zapatero A. (2006) Mortality among adult patients admitted to the hospital on weekends. Eur J Intern Med 17(5): 322–324. 16864005 10.1016/j.ejim.2006.01.003

[38] Becker DJ (2008) Weekend hospitalization and mortality: a critical review. Expert Rev Pharmacoecon Outcomes Res 8(1): 23–26. 10.1586/14737167.8.1.23 20528352

[39] Becker DJ (2007) Do hospitals provide lower quality care on weekends? Health Serv Res 42(4): 1589–1612. 17610439 10.1111/j.1475-6773.2006.00663.x PMC1955270

[40] Cram P, Hillis SL, Barnett M, Rosenthal GE (2004) Effects of weekend admission and hospital teaching status on in-hospital mortality. Am J Med 117(3): 151–157. 15276592 10.1016/j.amjmed.2004.02.035

[41] Carr BG, Reilly PM, Schwab CW, Branas CC, Geiger J, Wiebe DJ (2011) Weekend and night outcomes in a statewide trauma system. Arch Surg 146(7): 810–817. 10.1001/archsurg.2011.60 21422328

[42] McCarthy ML, Ding R, Pines JM, Zeger SL (2011) Comparison of methods for measuring crowding and its effects on length of stay in the emergency department. Acad Emerg Med 18(12): 1269–1277. 10.1111/j.1553-2712.2011.01232.x 22168190

[43] Locker TE, Mason SM (2005) Analysis of the distribution of time that patients spend in emergency departments. BMJ 330(7501): 1188–1189. 15843426 10.1136/bmj.38440.588449.AE PMC558017

[44] Wilber ST, Gerson LW (2003) A research agenda for geriatric emergency medicine. Acad Emerg Med 10(3): 251–260. 12615591 10.1111/j.1553-2712.2003.tb01999.x

[45] Retezar R, Bessman E, Ding R, Zeger SL, McCarthy ML (2011) The effect of triage diagnostic standing orders on emergency department treatment time. Ann Emerg Med 57(2): 89–99.e2. 10.1016/j.annemergmed.2010.05.016 20541284

[46] Weber EJ, Mason S, Freeman JV, Coster J (2012) Implications of England's four-hour target for quality of care and resource use in the emergency department. Ann Emerg Med 60(6): 699–706. 10.1016/j.annemergmed.2012.08.009 23102917

[47] Jones P, Schimanski K (2010) The four hour target to reduce Emergency Department 'waiting time': a systematic review of clinical outcomes. Emerg Med Australas 22(5): 391–398. 10.1111/j.1742-6723.2010.01330.x 20880296

[48] Mason S, Weber EJ, Coster J, Freeman J, Locker T (2012) Time patients spend in the emergency department: England's 4-hour rule-a case of hitting the target but missing the point? Ann Emerg Med 59(5): 341–349. 10.1016/j.annemergmed.2011.08.017 22088495

