# Supplementary material for: Organisational Factors Induce Prolonged Emergency Department Length of Stay in Elderly Patients – A Retrospective Cohort Study
Source: PLoS One. 2015 Aug 12;10(8):e0135066. doi: 10.1371/journal.pone.0135066 (PMC4534295; doi:10.1371/journal.pone.0135066)
Supplement: S1 Table — (DOCX) [file pone.0135066.s001.docx]

**Supporting Information**

**S1 Table. Miscellaneous complaints in elderly and younger patients and prolonged ED-LOS.**

| **Miscellaneous complaints** | **Elderly patients (n = 1782)** | | **Younger patients (n = 597)** | |
| --- | --- | --- | --- | --- |
|  | **Number of ED visits (%)** | **Prolonged ED-LOS (%)** | **Number of ED visits (%)** | **Prolonged ED-LOS (%)** |
| Musculoskeletal system | 51 (28.5%) | 16 (31.4%) | 15 (12.2%) | 4 (26.7%) |
| Genitourinary system | 51 (28.5%) | 18 (35.3%) | 12 (9.8%) | - |
| External causes | 39 (21.8%) | 6 (15.4%) | 57 (43.3%) | 5 (8.8%) |
| Injury and poisoning | 22 (12.3%) | 6 (15.4%) | 37 (30.1%) | 6 (6.2%) |
| Skin and subcutaneous tissue | 14 (7.8%) | 6 (42.9%) | 1 (0.8%) | - |
| Eye and adnexa | 2 (1.1%) | - | 1 (0.8%) | - |
| Ear and mastoid process | - | - | - | - |
